# Coronavirus Pandemic (SARS-COV-2): Pre-Exercise Screening Questionnaire (PESQ) for Telepresential Exercise

**DOI:** 10.3389/fpubh.2020.00146

**Published:** 2020-04-21

**Authors:** Leônidas de Oliveira Neto, Vagner Deuel de Oliveira Tavares, Felipe Barreto Schuch, Kenio Costa Lima

**Affiliations:** ^1^Department in Arts, Federal University of Rio Grande do Norte, Natal, Brazil; ^2^Laboratory of Hormonal Measurements, Department of Physiology and Human Behavior, Federal University of Rio Grande do Norte, Natal, Brazil; ^3^Department of Sports Methods and Techniques, Federal University of Santa Maria, Brazil; ^4^Department of Odontology, Federal University of Rio Grande do Norte, Natal, Brazil

**Keywords:** exercise telehealth, COVID-19, behavior, E-health, Questionnaire

## Introduction

The coronavirus pandemic (SARS-COV2) began in 2019 in Wuhan, Hubei Province, China (1). At the present moment, there is an exponential increase in both the number of people infected and the number of people dead. On the 31st of March, more than 30,000 people were reported to have died from COVID-19 (1). In fact, the World Health Organization (WHO) (1) announced on the 30th of January, 2020, that COVID-19 is a public health emergency of international interest, and, more recently, on March 11th, 2020, the WHO classified it as a pandemic (1).

The pandemic process of infectious diseases occurs in three stages: (a) imported cases, (b) local transmission, and (c) sustained community transmission. In an attempt to contain the speed of dissemination of the new virus between and within territories, the WHO (2020) adopted several health measures, based both on the evolution of the disease and the analysis of cases that occurred between countries in Asia and Europe. Such measures have so far ranged from social isolation, surveillance of cases coming from epidemic areas, and increasing public awareness to infection control in health facilities (2).

Among the health facilities that are very crowded and that can be a space of virus transmission, the gymnasium, and sports facilities stand out. Thus, in an attempt to reduce the scenarios of high virus transmission in Brazil, the Ministry of Health announced, on March 13th, guidelines to avoid the spread of the coronavirus, such as choosing to exercise outdoors instead of taking gymnastics classes in enclosed spaces (3).

This article has aimed to discuss the need for screening for the practice of physical exercise and to present The Pre-Exercise Screening Questionnaire (PESQ) (Table 1) for screening and identify people that are in a suitable place to start exercising during the COVID-19 pandemic.

**Table 1:** Pre-Exercise Screening Questionnaire (PESQ).

**Take the Pre-Exercise Screening Questionnaire (PESQ)**
Being physically active is very safe for most people. Some people, however, should consult their doctors before starting physical exercise, especially if they experience symptoms of fever for many days.
Answer **Yes** or **No** to the Following Questions 1) Do you feel a sore throat? 2) Do you feel cough and sputum production? 3) Do you feel fatigue? 4) Do you feel short of breath or difficulty breathing? 5) Do you feel fever >37.8°C? 6) Have you had fever for more than three days >37.8°C? 7) Have you had any contact with anyone who has been diagnosed or suspected of the new coronavirus?
**If You Answered Yes** If you answered yes to question number seven (Q-6) and/or number nine (Q-7), You should ask for a medical clearance along with information about specific for starting exercise.
**If You Answered No** If you answered no to all the PESQ questions, you can be reasonably sure that you can exercise safely and have a low risk of having any medical complications from exercise.

## Current Peak Body Recommendations

A large and recent epidemiological study carried out in China demonstrated in 1,099 confirmed cases in the laboratory that the clinical manifestations most commonly found in patients with COVID-19 were fever (88.7%), cough (67.8%), fatigue (38.1%), sputum production (33.4%), shortness of breath (18.6%), sore throat (13.9%), and headache (13.6%) (4). Concerning fever, a body temperature over 38°C is being used as a cutoff to direct patients to medical examination, followed by tests for SARS-CoV2 and, if necessary, isolation and adequate treatment (5). While fever and cough are the most present symptoms, other less prevalent respiratory (shortness of breath and sputum production) and gastrointestinal (diarrhea) symptoms can be observed (6–9), suggesting differences in the viral tropism of COVID-19 compared to influenza. At this moment, fever, excessive nasal mucosa discharge (rhinorrhea), and muscle pain are predominant characteristics in COVID-19 (4, 10). Thus, evaluating these signs and symptoms before the practice of physical activity should be considered. It is important to note that the infected do not present any symptoms during the incubation period, and the incubation period can last for 5.5 days on average (11). However, the symptoms appear on average of 11.5 after the infection (11). Thus, these symptoms should be questioned daily. For this reason, as determined by the ACSM (2020), those who do not present signs and symptoms of the disease should be able continue to exercise regularly.

COVID-19 spreads rapidly from human to human (12). Some studies have demonstrated that each person infected transmits, on average, to four other persons (13). For this reason, social distancing and isolation are required. Facing this new scenario of social isolation, exercise professionals have been using online technology to prescribe, and monitor exercise (14), such as mobile telephones messages, apps, email, video calls, or other internet-based strategies (15).

Scientific societies, such as the American College of Sports Medicine (ASCM) and the Brazilian Society of Exercise and Sports Medicine (BSESM), have released reports and guides to assist exercise professionals during the COVID-19 pandemic crisis (16). In these documents, societies have highlighted the positive impact of the regular practice of physical exercise on the improvement of the immunological system in humans, highlighting that physically active people have a lower risk of developing chronic-degenerative diseases, which is pertinent, as a those with affected by this are at higher risk if infected by SARS-COV2 (17). The BSESM and the ACSM defend the practice of regular physical activity as an adjuvant factor in combating morbidity and mortality associated with coronavirus.

Additionally, the BSESM have also created a report that indicates that, in the presence of signs compatible with respiratory infections, such as fever, cough, and dyspnea (shortness of breath), the practice of exercise should be suspended. A brief communication from (18) the American College of Sports Medicine suggests that, for those do not exhibit signs of symptoms, the specific recommendations for their age and group should be followed without restrictions or limitations during the COVID-19 pandemic. Therefore, people in isolation and with a positive diagnosis for COVID-19, but who are asymptomatic, should be able to continue the regular practice of physical activity following a moderate intensity. However, in the presence of symptoms (e.g., fever, cough, and dyspnea), the practice of physical activity should be interrupted and medical assistance sought (18). Therefore, it is important to the exercise professional to be aware of how to evaluate and screen for these symptoms to suggest who can exercise safely.

## Pre-exercise Screening

A more rigid screening was adopted by the Federal Council of Dentistry (CFO), which still uses the feverish state (>37.3°C) of the last 14 days to assess the possibility or not of care (14). Temperature evaluation seems to be a pertinent pre-participation evaluation. Although non-specific symptom, it is one of the main symptoms of COVID-19, and, during the pandemic, it presents as a method with good sensitivity and ability to identify potentially contaminated people. As an example, a study of 138 patients hospitalized with COVID-19 in Wuhan, China, documented that fever was present in 98.6% (136/138) of hospitalized patients. The other two patients who did not have fever were in intensive care unit beds, which may make us think of two situations: (a) they could have been medicated, which masks the result, and (b) the feverish state is not an indicator of the severity of the disease (19). However, restrictions based only on body temperature may not be sufficient and must be associated with other findings. Assessing the presence of pre-existing comorbidity is also extremely important.

It is also essential to question a retrospective view of the events that preceded the last days of the beginning of the practice of physical activity. For this reason, it is important to observe the presence of risk of exposure behaviors, such as trips abroad and/or contact with people suspected virus. This also applies to places where no community transmission has been declared. Considering that telepresential training has no limitations. However, these criteria should be associated with the declared health situation of the client/patient's place of residence. If the place of residence is related to community contagion, this will not make sense.

Finally, particularities, such as the level of functional capacity, must be followed according to the ACSM recommendations (20), particularly for elderly people (21). The need to perform physical activity is as evident as the need to guarantee the necessary security of its practitioners. Criteria to functional independence proposed by Katz et al. (22), for the advanced activities of daily life, should be questioned before release for proposed exercises. If a population group has a poor health status, it tends to worsen dramatically after the involvement of COVID-19. Thus, questioning their independence for advanced activities of daily life may provide some safety data to ensure a greater degree of autonomy to practice without the direct supervision of a professional.

Given the aforementioned challenges during this pandemic crisis, it is critical for people to exercise. For the purpose of doing it safely, the screening of COVID-19-related symptoms prior to exercise may help to identify those that are able to start exercising.

Therefore, we have developed a tool to screen for the main COVID-19 symptoms to ensure the safety of exercise prescription. The Pre-Exercise Screening Questionnaire (PESQ) aims to quickly detect symptoms of COVID-19 to assess a person's readiness for physical exercise. This is prudent to avoid the potential risk of exacerbation of respiratory symptoms when starting exercise. The PESQ has seven questions that should be answered with a yes or no. Item 1 measures sore throat. Item 2 measures cough and sputum production. Item 3 measures fatigue. Item 4 measures shortness of breath or difficulty of breathing. Item 5 and 6 are related to fever, and Item 7 is related to any contact with anyone who has been diagnosed or suspected of the new coronavirus.

The instrument has some advantages since it is easy, simple, and quick to apply on a large scale without additional costs. This is particularly important due to the negative change in the global economic situation and the rapid growth of this disease. Also, the questionnaire is important not only for the identification of individuals with a greater need for clinical examinations but also for the identification of those who do not need immediate tests.

It is worth mentioning some limitations of the present study. For example, the PESQ will not identify asymptomatic people. Another disadvantage is that it is reliant on the interpretation of the individual's signs and symptoms without making available a professional assessment. Finally, the PESQ does not eliminate the need for medical clearance and/or exercise testing in many individuals; this is a simple screen tool for screening for relevant COVID-19 symptoms.

## Author Contributions

LN: conceptualization, project administration, and writing—original draft preparation. VT: reviewing and editing. FS: reviewing and editing. KL: conceptualization, project administration, and writing—original draft preparation.

## Conflict of Interest

The authors declare that the research was conducted in the absence of any commercial or financial relationships that could be construed as a potential conflict of interest.

## References

[1] World Health Organization. Coronavirus Disease 2019 (?COVID-19). (2020). Available online at: https://www.who.int/docs/default-source/coronaviruse/situation-reports/20200226-sitrep-37-covid-19.pdf (accessed February 27, 2020).

[2] World Health Organization Novel Coronavirus (2019-nCoV). (2020). Available online at: https://www.who.int/emergencies/diseases/novel-coronavirus-2019/situation-reports

[3] Ministério de Saúde Saúde Anuncia Orientações Para Evitar a Disseminação Do Coronavírus. 2020 (2020). Available online at: https://www.saude.gov.br/noticias/agencia-saude/46540-saude-anuncia-orientacoes-para-evitar-a-disseminacao-do-coronavirus

[4] Guan W-J Ni Z-Y Hu Y Liang W-H Ou C-Q He J-X Clinical characteristics of coronavirus disease 2019 in China. N Engl J Med. (2020) 1–13. 10.1101/2020.02.06.20020974 32109013 PMC7092819

[5] World Health Organization Updated WHO Advice for International Traffic in Relation to the Outbreak of the Novel Coronavirus 2019-nCoV. (2020).

[6] Ren L-L Wang Y-M Wu Z-Q Xiang Z-C Guo L Xu T. Identification of a novel coronavirus causing severe pneumonia in human. Chin Med J. (2020). 10.1097/CM9.0000000000000722 32004165 PMC7147275

[7] Wang W Tang J Wei F. Updated understanding of the outbreak of 2019 novel coronavirus (2019-nCoV) in Wuhan, China. J Med Virol. (2020) 92:441–7. 10.1002/jmv.25689 31994742 PMC7167192

[8] Graham Carlos W Dela Cruz CS Cao B Pasnick S Jamil S. Novel Wuhan (2019-NCoV) coronavirus. Am J Respir Crit Care Med. (2020) 201:P7–8. 10.1164/rccm.2014P7 32004066

[9] Huang C Wang Y Li X Ren L Zhao J Hu Y. Clinical features of patients infected with 2019 novel coronavirus in Wuhan, China. Lancet. (2020) 395:497–506. 10.1016/S0140-6736(20)30183-5 31986264 PMC7159299

[10] Da Silva AA Ranieri TMS Torres FD Vianna FSL Paniz GR Sanseverino PB. Impact on pregnancies in south Brazil from the influenza A (H1N1) pandemic: Cohort study. PLoS ONE. (2014) 9:e88624. 10.1371/journal.pone.0088624 24558404 PMC3928255

[11] Stephen A. Lauer Kyra H. Grantz Qifang Bi Forrest K. Jones Qulu Zheng Hannah R. Meredith .The incubation period of coronavirus disease 2019 (COVID-19) from publicly reported confirmed cases: estimation and application. Ann Intern Med. (2020). 10.7326/M20-0504 PMC7081172 32150748

[12] Chan JFW Yuan S Kok KH To KKW Chu H Yang J. A familial cluster of pneumonia associated with the 2019 novel coronavirus indicating person-to-person transmission: a study of a family cluster. Lancet. (2020) 395:514–23. 10.1016/S0140-6736(20)30154-9 31986261 PMC7159286

[13] Liu Y Gayle AA Wilder-Smith A Rocklöv J. The reproductive number of COVID-19 is higher compared to SARS coronavirus. J Travel Med. (2020) 27:1–4. 10.1093/jtm/taaa021 32052846 PMC7074654

[14] de Odontologia CF Recomendações Para Atendimentos Odontológicos Em Tempos De COVID-19. (2020). Available online at: http://www.sciencemag.org/news/2020/02/paper-non-symptomatic-patient-transmitting-coronavirus-wrong (accessed 11 February, 2020).

[15] Gustavo Lima Isler e Afonso Antonio Machado *A d*í*ade treinador personalizado online e cliente: análise das relações interpessoais* (Tese). Univ Estadual Paul Inst Biociências Rio Claro (2015). p. 1–155.

[16] Leitão MB Lazzoli JK Torres FCMHL Informe Da Sociedade Brasileira De Medicina Do Exercício E Do Esporte (SBMEE) Sobre Exercício Físico E O Coronavírus (COVID-19). (2020). Available online at: http://website.cfo.org.br/wp-content/uploads/2020/03/Material-CDs-Coronavi%CC%81rus-CFO-1.pdf

[17] Pedersen BK Saltin B. Exercise as medicine - evidence for prescribing exercise as therapy in 26 different chronic diseases. Scand J Med Sci Sport. (2015) 25:1–72. 10.1111/sms.12581 26606383

[18] Joy L Staying Active During COVID-19. (2020). Available online at: https://www.exerciseismedicine.org/support_page.php/stories/?b=892

[19] Wang D Hu B Hu C Zhu F Liu X Zhang J Clinical characteristics of 138 hospitalized patients with 2019 novel coronavirus-infected pneumonia in Wuhan, China. J Am Med Assoc. (2020) 323:1061–9. 10.1001/jama.2020.1585 PMC7042881 32031570

[20] Garber CE Blissmer B Deschenes MR Franklin BA Lamonte MJ Lee IM. Quantity and quality of exercise for developing and maintaining cardiorespiratory, musculoskeletal, and neuromotor fitness in apparently healthy adults: guidance for prescribing exercise. Med Sci Sports Exerc. (2011) 43:1334–59. 10.1249/MSS.0b013e318213fefb 21694556

[21] Tomás MT Galán-Mercant A Carnero EA Fernandes B. Functional capacity and levels of physical activity in aging: a 3-year follow-up. Front Med. (2017) 4:244. 10.3389/fmed.2017.00244 29376052 PMC5767296

[22] Katz S Ford AB Moskowitz RW Jackson BA Jaffe MW. Studies of illness in the aged the index of ADL: a standardized measure of biological and psychosocial function. JAMA. (1963) 185:914–9. 10.1001/jama.1963.03060120024016 14044222

